# Distribution of Beijing Genotype Among Clinical Isolates of *M. tuberculosis* Circulating in Kazakhstan

**DOI:** 10.5195/cajgh.2014.145

**Published:** 2014-12-12

**Authors:** Ainur Akhmetova, Venera Bismilda, Leila Chingissova, Ulan Kozhamkulov

**Affiliations:** 1 Center for Life Sciences, Nazarbayev University, Astana, Kazakhstan; 2 Department of Bacteriology, National Center for Tuberculosis Problems, Almaty, Kazakhstan

**Keywords:** tuberculosis, mycobacteria, genotyping, Kazakhstan, Beijing genotype

## Abstract

**Introduction:**

Methods of genotyping of M. tuberculosis play an important role in tuberculosis (TB) infection control. These techniques are used to detect or exclude laboratory errors, control recurrent cases, and determine ways of TB transmission. Today, there are more than 10 methods of genotyping; MIRU-VNTR is one of the most widely used methods in the world. In this study we aimed to estimate biological diversity of clinical isolates of M. tuberculosis from different regions of Kazakhstan based on MIRU-VNTR analysis.

**Materials and methods:**

MIRU-VNTR was used to genotype 134 clinical isolates of M. tuberculosis isolated from new cases and recurrent cases of TB from different regions of Kazakhstan. Amplification was done using 15 MIRU-VNTR loci. Determination of the number of tandem repeats in the corresponding locus was performed via Quantity One v.4.4.0 (BioRad, USA) software. H37Rv (NC_000962) reference strain was used as a positive control.

**Results:**

Phylogenic tree was built using www.miru-vntr.org web-resource based on the results of MIRU-VNTR analysis. Beijing family strains associated with drug resistance to antituberculosis drugs were prevalent among all isolates of M. tuberculosis circulating in Kazakhstan. Strains of the Beijing genotype were prevalent in both new cases (65%) and recurrent cases (89.4%) of tuberculosis. The second meaningful genotype that is spread in the territory of Kazakhstan is LAM, the frequency of distribution is 7.3% in new and 4.5% in recurrent cases. Other families of M. tuberculosis such as Ural, Haarlem, CAS, NEW-1, S were found in less than 4% of cases.

**Conclusion:**

Prevalence of Beijing family strains among all isolates of M. tuberculosis from different regions of Kazakhstan was shown. Strains of this family are prevalent among young people. This genotype is responsible for ongoing TB transmission in the present time. This genotype is more virulent; therefore, investigation of the epidemiology of the Beijing genotype plays crucial role in the monitoring of tuberculosis.

